# Leukotriene receptor antagonists enhance HCC treatment efficacy by inhibiting ADAMs and suppressing MICA shedding

**DOI:** 10.1007/s00262-020-02660-2

**Published:** 2020-07-18

**Authors:** Jun Arai, Kaku Goto, Yumi Otoyama, Yoko Nakajima, Ikuya Sugiura, Atsushi Kajiwara, Masayuki Tojo, Yuki Ichikawa, Shojiro Uozumi, Yuu Shimozuma, Manabu Uchikoshi, Masashi Sakaki, Hisako Nozawa, Ryo Nakagawa, Ryosuke Muroyama, Naoya Kato, Hitoshi Yoshida

**Affiliations:** 1 Division of Advanced Genome Medicine, The Institute of Medical Science, The University of Tokyo, Tokyo, Japan; 2 Division of Gastroenterology, Department of Medicine, Showa University School of Medicine, 1-5-8 Hatanodai, Shinagawa-ku, Tokyo, 142-8666 Japan; 3 Department of Gastroenterology, Graduate School of Medicine, Chiba University, Chiba, Japan

**Keywords:** A disintegrin and metalloprotease 9, Hepatocellular carcinoma, MHC class I-related chain A, Regorafenib, Leukotriene D4

## Abstract

In our previous genome-wide association study, we demonstrated the association between MHC class I-related chain A (MICA) and hepatocellular carcinoma (HCC) development in patients with chronic hepatitis C. Increasing membrane-bound MICA (mMICA) in cancer cells by reducing MICA sheddases facilitates natural killer (NK) cell-mediated cytotoxicity. Our recent study clarified that A disintegrin and metalloproteases (ADAM), including ADAM9, are MICA sheddases in HCC, and that the suppression of ADAMs increases mMICA, demonstrating the rationality of mMICA-NK targeted therapy. Furthermore, we showed that regorafenib suppresses ADAM9 transcriptionally and translationally. A library of FDA-approved drugs was screened for more efficient inhibitors of ADAM9. Flow cytometry evaluation of the expression of mMICA after treatment with various candidate drugs identified leukotriene receptor antagonists as potential ADAM9 inhibitors. Furthermore, leukotriene receptor antagonists alone or in combination with regorafenib upregulated mMICA, which was in turn downregulated by leukotriene C4 and D4 via ADAM9 function. Our study demonstrates that leukotriene receptor antagonists could be developed as novel drugs for immunological control and suppression of ADAM9 in HCC. Further, leukotriene receptor antagonists should be explored as combination therapy partners with conventional multi-kinase inhibitors for developing therapeutic strategies with enhanced efficacies for HCC management and treatment.

**Electronic supplementary material:**

The online version of this article (10.1007/s00262-020-02660-2) contains supplementary material, which is available to authorized users.

## Introduction

Even with advancements in cancer treatment and management, hepatocellular carcinoma (HCC) remains one of the most common causes of cancer-related deaths worldwide. HCC could develop from various liver diseases, including chronic hepatitis and liver cirrhosis [1]. In Japan, hepatitis B or C viral infection is a major cause of HCC [2]. Although clinical procedures such as radiofrequency ablation and transarterial chemoembolization provide excellent local treatment and extend overall survival, sorafenib and lenvatinib, which were approved for the treatment of HCC as first-line chemotherapy in 2018 [3], are the only available systemic drugs for HCC. Therefore, the identification and development of new and better drugs are required for the efficient management of HCC.

Recently, the multi-kinase inhibitor regorafenib has shown better potency in the Phase 3 RESORCE (Regorafenib after sorafenib in patients with hepatocellular carcinoma) trial that enrolled patients with sorafenib-resistant HCC [4]. Regorafenib is a sorafenib analog approved as a second-line therapy to treat colon cancer and pancreatic neuroendocrine tumors and is known to disrupt angiogenesis and the tumor microenvironment [5–7].

In the tumor microenvironment, an active innate immunity is critical for eliminating cancer cells and preventing disease recurrence and metastasis [8]. Cancer immunoediting is an extrinsic tumor suppressor mechanism that engages after cellular transformation has occurred, and intrinsic tumor suppressor mechanisms have failed. During its elimination phase, innate and adaptive immunity work together to prevent tumors from developing before they become clinically apparent [8]. NK ligands, such as MHC class I polypeptide-related sequence A (MICA) binds to activating receptors on innate immune cells, leading to the release of pro-inflammatory and immunomodulatory cytokines, which in turn establish a microenvironment that facilitates the development of a tumor-specific adaptive immune response [9].

In our previous genome-wide association study (GWAS), MICA was identified as an HCC susceptibility gene [10]. MICA is an NK group D (NKG2D) ligand expressed on the surface of infected or cancerous cells for elimination by NK cells. We also showed that the restoration of membrane-bound MICA (mMICA) expression augmented NK cell-mediated anti-HCC cytotoxicity [11]. In this prior study, the increased expression of MICA specific to HCC cells enhanced NK cell-mediated cytotoxicity in co-culture, which was further reinforced by treatment with an inhibitor of MICA sheddase. Similarly, the augmented anti-tumor activity of NK cells via NKG2D was observed in vivo. Importantly, mMICA is subject to proteolytic shedding, and the released soluble MICA (sMICA) is an immunological decoy, in the serum. A disintegrin and metalloprotease (ADAM) and matrix metalloprotease (MMP) have been shown to shed mMICA in several cancer cell lines [12].

Sorafenib enhanced NK cell cytotoxicity to HCC by downregulating the expression of ADAM9, a protease responsible for mMICA shedding [13], and ADAM10 [14]. Recently, we discovered that regorafenib upregulates mMICA to a greater extent than sorafenib, suppressing ADAM9 in hepatoma cells [15], confirming the role of ADAM9 as an immunotherapeutic target. In this study, we aimed to identify new inhibitors of ADAM9 from a library of FDA-approved drugs in vitro. In addition, the molecular effects and immunotherapeutic impacts of the positive hits were analyzed in HCC cells.

## Materials and methods

### Cells, reagents, and antibodies

Sorafenib and regorafenib were obtained from Selleck Chemicals (Houston, TX, USA) and Cell Signaling Technology (Danvers, MA, USA), respectively. Ilomastat, leukotriene C4/D4, and pranlukast /montelukast were purchased from Selleck (Houston, TX, USA), Cayman CHEMICAL (Houston, TX, USA), and TCI (Tokyo, Japan), respectively. Cell Counting Kit-8 (CCK8) was purchased from Dojindo (Kumamoto, Japan). The FDA-Approved Drug Screen-well library was obtained from Enzo Life Sciences (Farmingdale, NY, USA). HepG2 and PLC/PRF/5 cells were obtained from American Type Culture Collection (Manassas, VA, USA) and cultured according to the supplier’s protocols. The cell lines were authenticated by short tandem repeat analysis (Bex, Tokyo, Japan) in January 2018. ADAM9 siRNA was purchased from Dharmacon (Ann Arbor, Michigan, USA).

### Cell viability assays

HepG2 and PLC/PRF/5 cells (2 × 10^5^ cells/mL/well) were plated in 24-well plates and incubated at 37 °C for 24 h. The cells were then treated with ilomastat, leukotriene C4/D4, pranlukast, montelukast, or regorafenib for 48 h. After the treatment, the culture supernatant was removed, and cell viability was measured using the CCK8 assay kit (Dojindo). Briefly, 1 ml CCK-8 reagent diluted following the manufacturer’s instructions was added per well and the plates were incubated at 37 °C for 1 h. After incubation, absorbance at 450 nm was measured using a microplate reader to determine the number of viable cells’.

### ELISA

The concentration of sMICA in the PLC/PRF/5 and HepG2 cell culture supernatants were assessed using a MICA ELISA Kit (Diaclone, Besançon, France) as described previously [15].

### Flow cytometry

Three-milliliters of suspended hepatoma cells (2 × 10^5^ cells/mL) were added to each well of a 6-cm dish. After incubating for 24 h at 37 °C, the cells were treated with ilomastat, leukotriene C4/D4, pranlukast, montelukast, or regorafenib for 48 h. The cells were then collected and incubated with Alexa Fluor 488-conjugated mouse IgG2B isotype control or Alexa Fluor 488-conjugated human MICA antibody (R&D Systems, Minneapolis, MN, USA) following the manufacturer’s protocol. Fluorescent signals were detected using a BD Accuri C6 flow cytometer (BD Biosciences, San Jose, CA, USA). Statistical information of flow cytometry is shown in (Supplementary Table 1).

### Quantitative reverse transcription-polymerase chain reaction (qRT-PCR)

Relative mRNA levels were quantified as previously described [15] using the following primer sets:

MICA-F: 5′-CTTCCTGCTTCTGGCTGGCATC-3′,

MICA-R: 5′-CAGGGTCATCCTGAGGTCCTTTC-3′,

ADAM9-F: 5′-AAGAATTGTCACTGTGAAAATGGCT-3′,

ADAM9-R: 5′-CATTGTATGTAGGTCCACTGTCCAC-3′,

ADAM10-F: 5′-ACGGAACACGAGAAGCTGTG-3′,

ADAM10-R: 5′-CCGGAGAAGTCTGTGGTCTG-3′,

ADAM17-F: 5′-GTCGAGCCTGGCGGTAGAATCTTC-3′,

ADAM17-R: 5′-CTCCACCTCTCTGGGCAGCCTTC-3′,

GAPDH-F: 5′-ATGGGGAAGGTGAAGGTCG-3′,

GAPDH-R: 5′-GGGGTCATTGATGGCAACAATA-3′.

### In vitro ADAM9 assay

Recombinant human ADAM9 (R&D systems; 20 µg/mL) was incubated with a fluorescent peptide substrate (BioZyme, NC, USA; 10 μM) in the presence of DMSO or individual compounds, following the manufacturer’s instructions. A library of FDA-approved drugs was tested for enzymatic inhibition of ADAM9, with ilomastat as the ADAM9 control inhibitor. After incubating for 2 h at 37 °C in opaque black plates, the fluorescent signals (*λ* excitation = 485 nm, *λ* emission = 530 nm), and the relative enzymatic activities were calculated.

### Statistical analyses

All values presented indicate the mean and standard error of the mean (SEM) unless otherwise indicated. Differences in the expression of mMICA between controls and treated samples were determined using Dunnett’s test. Differences of sMICA levels between treatment groups and control groups were determined using paired, two-tailed Student’s *t* test. *P* values less than 0.05 were considered statistically significant.

## Results

### ADAM9 inhibition suppressed MICA shedding

**Fig. 1:**
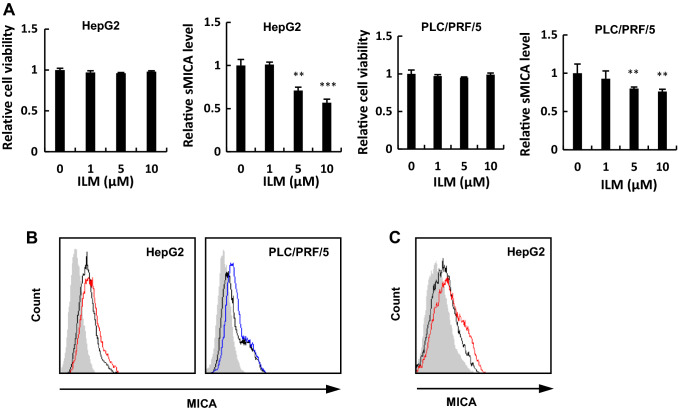
ADAM9 inhibitor, ilomastat, decreased sMICA secretion by HCC cells. **a** PLC/PRF/5 and HepG2 cells were treated with ilomastat for 48 h and the cell viabilities and sMICA levels were determined by CCK8 assay and ELISA, respectively. HepG2 and PLC/PRF/5 cells were treated with ilomastat (**b**) (no treatment in black and HepG2 and PLC/PRF/5 treated with ilomastat in red and in blue, respectively) or siRNA against ADAM9 (**c**) (siCtrl and siADAM9 in black and red, respectively) for 48 h and mMICA level was assessed by flow cytometry; the isotype controls are shown as gray histograms. Fluorescence intensity and cell counts are indicated on the X and Y axis, respectively. **P < 0.01; ***P < 0.005. Error bars represent SEM. Representative data from three independent experiments with consistently similar results are shown. *ILM* ilomastat

To identify the relationship between ADAM9 and mMICA shedding in HCC cells, HepG2 and PLC/PRF/5 cells were treated with ilomastat, an ADAM9 inhibitor. Ilomastat treatment decreased the sMICA levels by more than 40% compared to that in the control, with no observable cytotoxicity (Fig. 1a). Furthermore, ilomastat treatment restored mMICA in HepG2 and PLC/PRF/5 cells (Fig. 1b).

A previous study reported a 70% decrease in sMICA in the supernatant of ADAM9 siRNA (siADAM9)-transfected cells [15]. In our study, the upregulation of mMICA was confirmed by siADAM9 treatment in HepG2 cells (Fig. 1c). Meanwhile, ilomastat did not affect the mRNA expression of MICA or ADAM9. In addition, levels of ADAM10 and ADAM17, the known MICA sheddases in HCC, remained unaffected (Supplementary Fig. 1a) [13–16].

### Leukotriene receptor antagonists inhibited ADAM9 activity in vitro

**Fig. 2:**
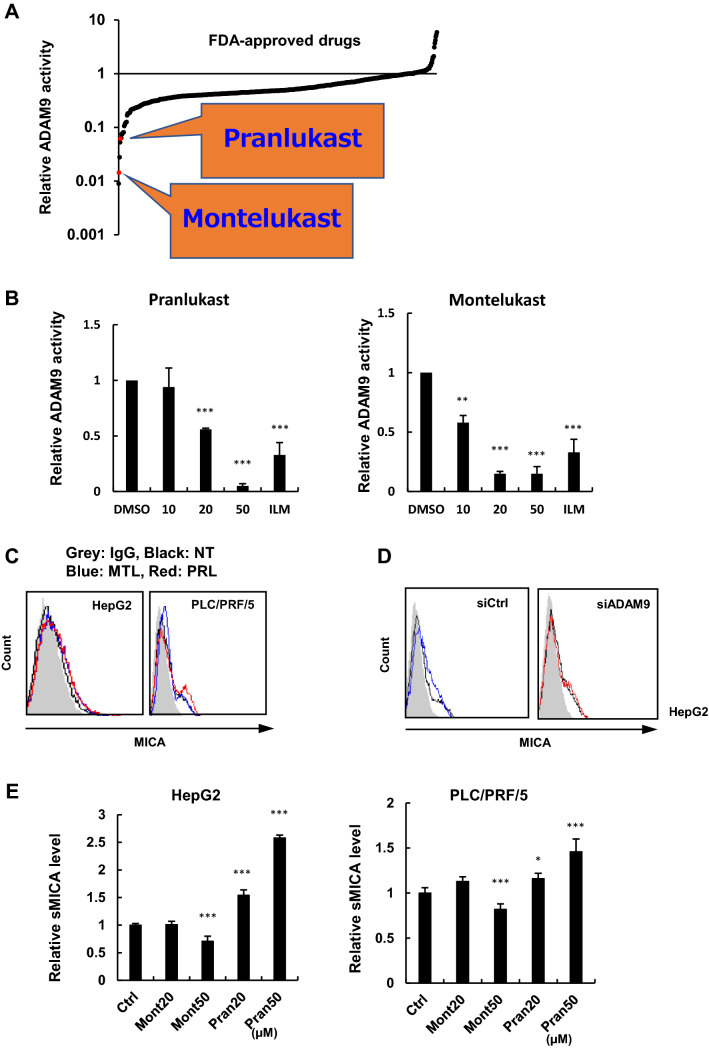

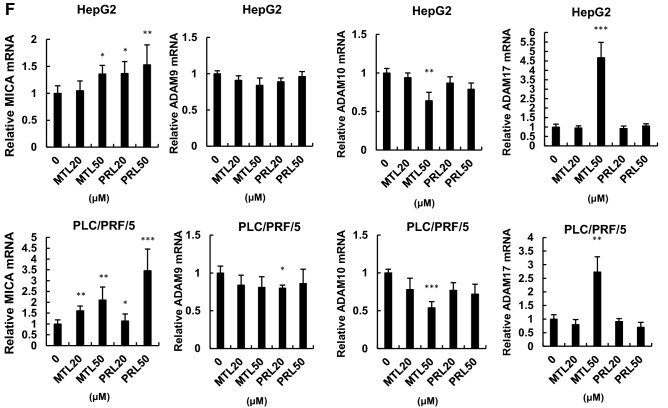
Leukotriene receptor antagonists inhibited ADAM9 in vitro and enhanced mMICA level in HCC cells. **a** Inhibitory effects of approved drugs on ADAM9 in vitro. **b** Enzymatic inhibition of ADAM9 by pranlukast or montelukast in vitro. **c** After treatment with pranlukast and montelukast, mMICA expression was analyzed by flow cytometry in HepG2 and PLC/PRF/5 cells; mMICA expression increased after 48 h treatment with no treatment, 50 µM montelukast, and 50 µM pranlukast in black, blue, and, red, respectively. The isotype controls are shown as gray histograms, and fluorescence intensity and cell counts are indicated on the X and Y axis, respectively. **d** The effects of pranlukast on mMICA expression in HepG2 cells were examined in the presence of siCtrl (0 and 50 µM in black and blue, respectively) or siADAM9 (0 and 50 µM shown in black and red, respectively) by flow cytometry. The isotype controls are shown as gray histograms, and fluorescence intensity and cell counts are indicated on the X and Y axis, respectively. **e** sMICA levels were determined by CCK8 assay and ELISA, respectively, after treatment with pranlukast and montelukast in HepG2 and PLC/PRF/5 cells. **f** Relative mRNA levels of MICA and ADAMs were analyzed by qRT-PCR after pranlukast / montelukast treatment. *P < 0.05; **P < 0.01; ***P < 0.005. Error bars represent SEM. Representative data from three independent experiments with consistently similar results are shown

We recently established a new in vitro system to evaluate ADAM9 activity similar to our previous assay system for ADAM17 [17]. An in vitro screen using a library of FDA-approved drugs identified that leukotriene receptor antagonists, pranlukast, and montelukast, dramatically suppressed the enzymatic activity of ADAM9, at 34 μM and 41 μM concentrations, respectively (Fig. 2a). The in vitro assays confirmed that both pranlukast and montelukast inhibited ADAM9 in a dose-dependent manner (Fig. 2b). However, pranlukast and montelukast did not suppress the enzymatic activities of known MICA sheddases in HCC ADAM10 and ADAM17, in vitro (Supplementary Fig. 1b).

### Leukotriene receptor antagonists elevated mMICA levels

Next, we tested the effects of pranlukast and montelukast on mMICA in HepG2 and PLC/PRF/5 cells. A 48 h treatment with 50 µM pranlukast or montelukast increased mMICA expression in HepG2 cells (Fig. 2c) statistically significantly (Supplementary Table 1). The same tendency was observed in PLC/PRF/5 (Fig. 2c) without significance in this setting (Supplementary Table 1).

This leukotriene receptor antagonist treatment-induced increase in mMICA was canceled in the presence of si-ADAM9 in HepG2 (Fig. 2d). A similar trend, though weaker in a limited fashion without reaching statistical significance, was observed consistently in PLC/PRF/5. These treatments did not induce any cytotoxicities in either cell line (Supplementary Fig. 1c).

Further, the effects of leukotriene receptor antagonists on sMICA concentration were examined in HepG2 and PLC/PRF/5 cells. Interestingly, montelukast treatment suppressed sMICA levels, while pranlukast treatment increased the levels of sMICA in HepG2 and PLC/PRF/5 cells (Fig. 2e). Furthermore, montelukast and pranlukast treatments increased the mRNA expression of MICA; and, treatment with 50 µM montelukast decreased ADAM10 and increased ADAM17 mRNA levels (Fig. 2f). The changes in the transcriptional levels and enzymatic activity of MICA and ADAM after treatment with montelukast/ pranlukast are shown in (Supplementary Table 2).

### Leukotriene D4 increased sMICA release and decreased mMICA levels

**Fig. 3:**
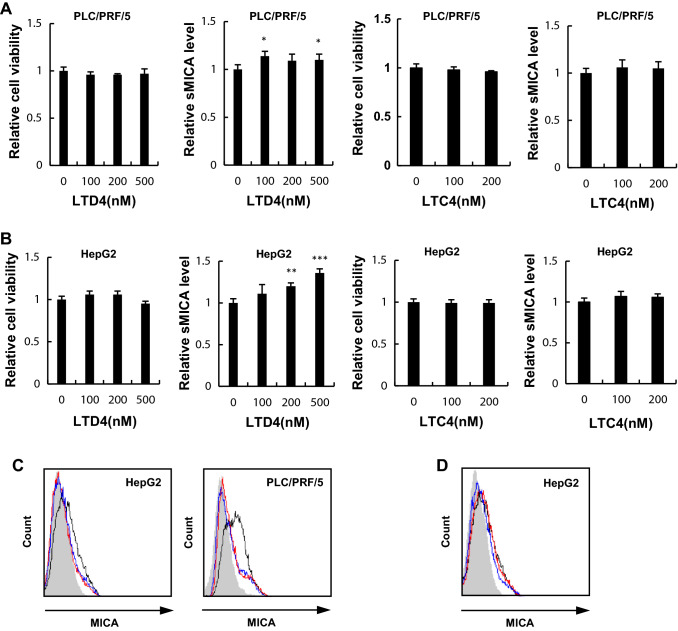
Impact of leukotriene C4/D4 on sMICA production by shedding mMICA via ADAM9 in HCC cells. Cell viabilities (**a**) and sMICA levels (**b**) were determined by CCK8 assay and ELISA, respectively, after treating HepG2 and PLC/PRF/5 with leukotriene C4/D4. (**c**) HepG2 and PLC/PRF/5 cells were treated with 100 µM leukotriene C4/D4 for 96 h, and mMICA level was assessed by flow cytometry. The isotype controls are shown as gray histograms (no treatment, leukotriene C4, and leukotriene D4 shown in black, blue, and red, respectively), and fluorescence intensity and cell counts are indicated on the X and Y axis, respectively. **d** The effect of leukotriene D4 for 48 h on mMICA level in HepG2 cells was examined in the presence of siCtrl (0 and 100 µM in black and blue, respectively) or siADAM9 (100 µM shown in red) by flow cytometry. The isotype controls are shown as gray histograms and fluorescence intensity and cell counts are indicated on the X and Y axis, respectively. *P < 0.05; **P < 0.01; ***P < 0.005. Error bars represent SEM. Representative data from three independent experiments with consistently similar results are shown

Pranlukast and montelukast are known to block leukotriene C4 and leukotriene D4 [18]. Among multiple molecules in the arachnoid acid- leukotriene pathway, the suppressive effect of pranlukast and montelukast is most potent against leukotriene D4, followed by leukotriene C4 [18]. Next, we investigated the influence of leukotriene C4/D4 on sMICA levels. Treatment with leukotriene C4 and leukotriene D4 did not induce cytotoxicity (Fig. 3a). Further, treatment with leukotriene D4 increased sMICA levels in HepG2 cells, while leukotriene C4 treatment did not increase sMICA (Fig. 3b). The mRNA levels of ADAM9, ADAM10, and ADAM17, as well as MICA, were not changed (Supplementary Fig. 1D, E).

In addition, leukotriene C4/D4 decreased mMICA levels in HepG2 and PLC/PRF/5 cells (Fig. 3c). However, the downregulation of mMICA by leukotriene D4 was abrogated by the knockdown of ADAM9 in HepG2 cells (Fig. 3d) with a limited impact as statistical significance was not achieved.

### Combination treatment of leukotriene receptor antagonists and clinical multi-kinase inhibitors (MKIs)

**Fig. 4:**
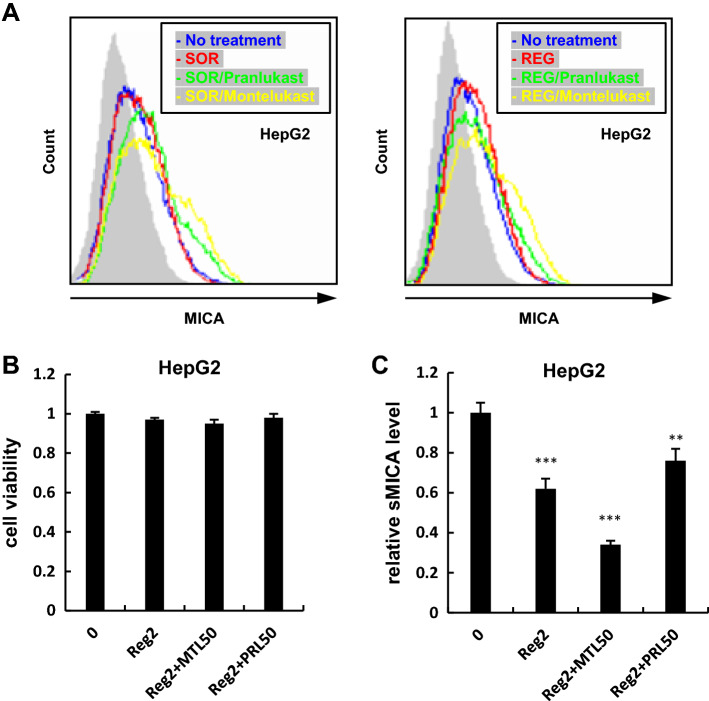
Combination treatments of leukotriene receptor antagonists and MKIs further enhanced mMICA. **a** After treating HepG2 with pranlukast / montelukast and sorafenib, or regorafenib, mMICA expression was analyzed by flowcytometry: no treatment, 2 µM of sorafenib/regorafenib, sorafenib/regorafenib and pranlukast, sorafenib/regorafenib and montelukast in blue, red, green, and yellow, respectively, and the isotype controls shown as gray histograms. Fluorescence intensity and cell counts are indicated on the X and Y axis, respectively. Cell viabilities (**b**) and sMICA levels (**c**) were determined by CCK8 assay and ELISA. **P < 0.01; ***P < 0.005. Error bars represent SEM. Representative data from three independent experiments with consistently similar results are shown. *SOR* sorafenib, *REG* regorafenib

We and others have reported that the currently approved MKIs against HCC, SOR, as well as REG, transcriptionally inhibits ADAM9 [13, 15], whose concurrent enzymatic inhibition by leukotriene receptor antagonists is therefore presumed to further enhance the expression of mMICA. To examine this hypothesis, we treated HepG2 cells for 48 h with 2 µM MKIs and leukotriene receptor antagonists. As hypothesized, the combination treatments enhanced mMICA levels more than either monotherapies (Fig. 4a) while the impacts were limited without achievement of statistical significance. The same trend, though less pronounced, was observed in PLC/PRF/5 cells (data not shown). In addition, in accordance with mMICA levels, the combination treatment with REG and montelukast decreased sMICA levels in HepG2 cells more than that with REG monotherapy and did not show significant cytotoxicity (Fig. 4b, c).

## Discussion

The concept of cancer immunoediting and the contribution of innate immunity are now recognized to be indispensable to eliminate cancer cells [8]. In the elimination phase of cancer immunoediting, MICA binds to activating receptors on NK cells, leading to the release of pro-inflammatory and immunomodulatory cytokines. NK cells are highly accumulated in the human liver, representing 30–50% of all hepatic lymphocytes and activate NK cells through the MICA-NKG2D system [19]. Suppression of ADAM9 activity enhanced the NK cell cytotoxicity against HCC by upregulating mMICA [13, 15]. ADAM9 has also been reported to be overexpressed in the cancer microenvironment of several cancer types, including liver cancer [20–24].

In this study, we discovered that leukotriene receptor antagonists, pranlukast, and montelukast, suppress ADAM9 activity in vitro (Fig. 2a, b), indeed increasing mMICA levels in HepG2 and PLC/PRF/5 cells (Fig. 2c) without cytotoxic effects (Supplementary Fig. 1c). The effect via direct targeting of ADAM9 was confirmed by siADAM9-mediated partial abrogation of the leukotriene receptor antagonist-induced upregulation of mMICA (Fig. 2d). Our previous and current studies demonstrated the upregulation of mMICA after ADAM9 downregulation [15] and ADAM9 inhibition (Fig. 1b), suggesting that ADAM9 contributes to the shedding of mMICA.

Notably, ADAM9 is acknowledged as a putative therapeutic target in HCC owing to its role in the immune microenvironment and cancer development [20]. Previous studies have demonstrated the relationship between ADAM9 expression and clinicopathological features, including disease prognosis, shortens overall survival, tumor grade, metastasis, and the development of resistance in various cancers, including HCC [20]. One potential mechanism by which ADAM9 expression affects the overall survival of HCC patients is its involvement in MICA shedding [13, 15]. Also Dengdi et al. reported that the miR-488, targeting ADAM9, is negatively associated with tumor size, and shorter overall survival in HCC patients, as a tumor suppressor and a potential therapeutic target [25]. In addition, ADAM9 was recently reported to mediate IL-6 induced epithelial-mesenchymal transition (EMT), resulting in IL-6 induced HCC cell migration and invasion [26].

Two different approaches could be used to inhibit ADAM9 in cancer treatment. First, as with sorafenib/regorafenib, ADAM9 function could be inhibited by decreasing its transcription levels [13, 15]. Second, the enzymatic activity of ADAM9 could be suppressed, which was achieved by using leukotriene receptor antagonists in this study. Treatment with pranlukast induced the expression of MICA at the transcription level more than that with montelukast treatment (Fig. 2e, f). Increased transcriptional expression of MICA generally increases sMICA as well as mMICA as the amount of sMICA is correlated with mMICA level when the shedding efficiency is unchanged.

In this study, treatment with montelukast, but not with pranlukast, significantly decreased sMICA concentration (Fig. 2e), presumably due to the structural differences between montelukast and pranlukast. As a result, montelukast suppressed the function of ADAM9 and ADAM10 enzymatically and transcriptionally, respectively. At the same time, the transcriptional level of ADAM17 was increased (Fig. 2b, f), indicating the balance of MICA sheddases in HCC, which was reported in our previous study [16]. As we previously tested, PLC/PRF/5 and HepG2 cells transfected siRNAs against ADAM9, ADAM10, and ADAM17 showed significantly decreased sMICA levels [17]. Although montelukast increased mRNA levels of ADAM17 and MICA, it significantly decreased ADAM10 mRNA and ADAM9 enzymatic activity. As a result, sMICA production was suppressed due to the reduced activity of ADAM9 and ADAM10, as shown in Supplementary Table 2. Interestingly, combination therapy with a transcriptional inhibitor and an enzymatic inhibitor induced a much higher increase in mMICA levels than the monotherapies, potentially due to the enhanced anti-HCC activity of NK cells that was previously demonstrated in vitro and in vivo [11].

As mentioned above, treatment with 100 µM leukotriene C4/D4 for 96 h decreased mMICA expression in HepG2 cells (Fig. 3c) more than that with the 48 h treatment (Fig. 3d). Furthermore, treatment with siADAM9 abrogated the decrease in mMICA in cells treated with 100 µM leukotriene C4/D4; since treatment with leukotriene C4/D4 increased sMICA, leukotriene C4/D4 induced MICA shedding via ADAM9 (Fig. 3d). Since leukotriene D4 decreased mMICA levels in HepG2 and PLC/PRF/5 cells and the downregulation of mMICA by leukotriene D4 was abrogated by the knockdown of ADAM9 in HepG2 cells (Fig. 3d), leukotriene D4 enhances mMICA shedding through ADAM9. Collectively, leukotriene signaling was indicated to be involved in the regulation of ADAM9.

Furthermore, Zhou et al. reported that circulating leukotriene D4 in HCC patients is significantly higher than that in healthy subjects, and may have a role in the pathogenesis of HCC [27]. Leukotriene D4 is widely considered to induce tumor proliferation and correlate negatively with patient survival in colon cancer [28–30]. Further studies on the molecular genetics of ADAM9, the tumor microenvironment, and cancer metabolism are required to understand the pro-tumoral effects of leukotriene D4.

These data indicate the potential for suppression of leukotriene C4 and leukotriene D4 with leukotriene receptor antagonists to enhance the elimination of cancer cells by NK cells through ADAM9 inhibition, and subsequent inhibition of MICA shedding, potentially upregulating MICA expression. The improved potency of leukotriene receptor antagonists emphasizes the significance of ADAM9 in HCC progression and suggests that leukotrienes may be important druggable targets to boost mMICA and restore innate immunity against HCC. Further studies are required to confirm that the restoration of MICA by the leukotriene receptor antagonists improves tumor-killing.

Following our discovery in vitro, open questions regarding the chemical mode of inhibition of ADAM9 by leukotriene antagonists, the mechanism of ADAM9 regulation by leukotriene signaling, immunotherapeutic efficacy of the leukotriene receptor antagonists combined with MKIs and related molecular as well as therapeutic aspects remain. All these intriguing viewpoints to be considered in upcoming studies will uncover the mechanistic link between leukotriene signaling and ADAM9 and will develop methods of pharmacological modulation.

Recently, regorafenib was reported to improve the outcome in HCC patients with sorafenib-resistant disease in the RESORCE trial [4]. This was possibly explained by our previous study indicating that regorafenib potentiates immune-mediated HCC cell death by promoting mMICA expression to a greater extent than sorafenib, by mainly targeting ADAM9 [15]. Again, our results would serve to develop a critical strategy to identify new treatment options for HCC. Importantly, leukotriene receptor antagonists could be an attractive agent for the immunological control of HCC, which also suppresses ADAM9 enzymatically, resulting in increased treatment efficacy when used in combination with conventional MKIs.

## Electronic supplementary material

Below is the link to the electronic supplementary material.

Supplementary file1 (PPTX 147 kb)

Supplementary file2 (PDF 432 kb)

Supplementary file3 (PDF 69 kb)

Supplementary file4 (PDF 25 kb)

Supplementary file5 (PDF 27 kb)

Supplementary file6 (DOCX 29 kb)

## Abbreviations

ADAM: A disintegrin and metalloprotease
CCK8: Cell Counting Kit-8
HCC: Hepatocellular carcinoma
MICA: MHC class I polypeptide-related sequence A
MKIs: Multi-kinase inhibitors.
mMICA: Membrane-bound MICA
MMP: Matrix metalloprotease
NK: Natural killer
SEM: Standard error of the mean
sMICA: Soluble MICA
