# Supplementary material for: Leukotriene receptor antagonists enhance HCC treatment efficacy by inhibiting ADAMs and suppressing MICA shedding
Source: Cancer Immunol Immunother. 2020 Jul 18;70(1):203–13. doi: 10.1007/s00262-020-02660-2 (PMC7838147; doi:10.1007/s00262-020-02660-2)
Supplement: Supplementary file 1 — Supplementary file1 (PPTX 147 kb) [file 262_2020_2660_MOESM1_ESM.pptx]

## Slide 1
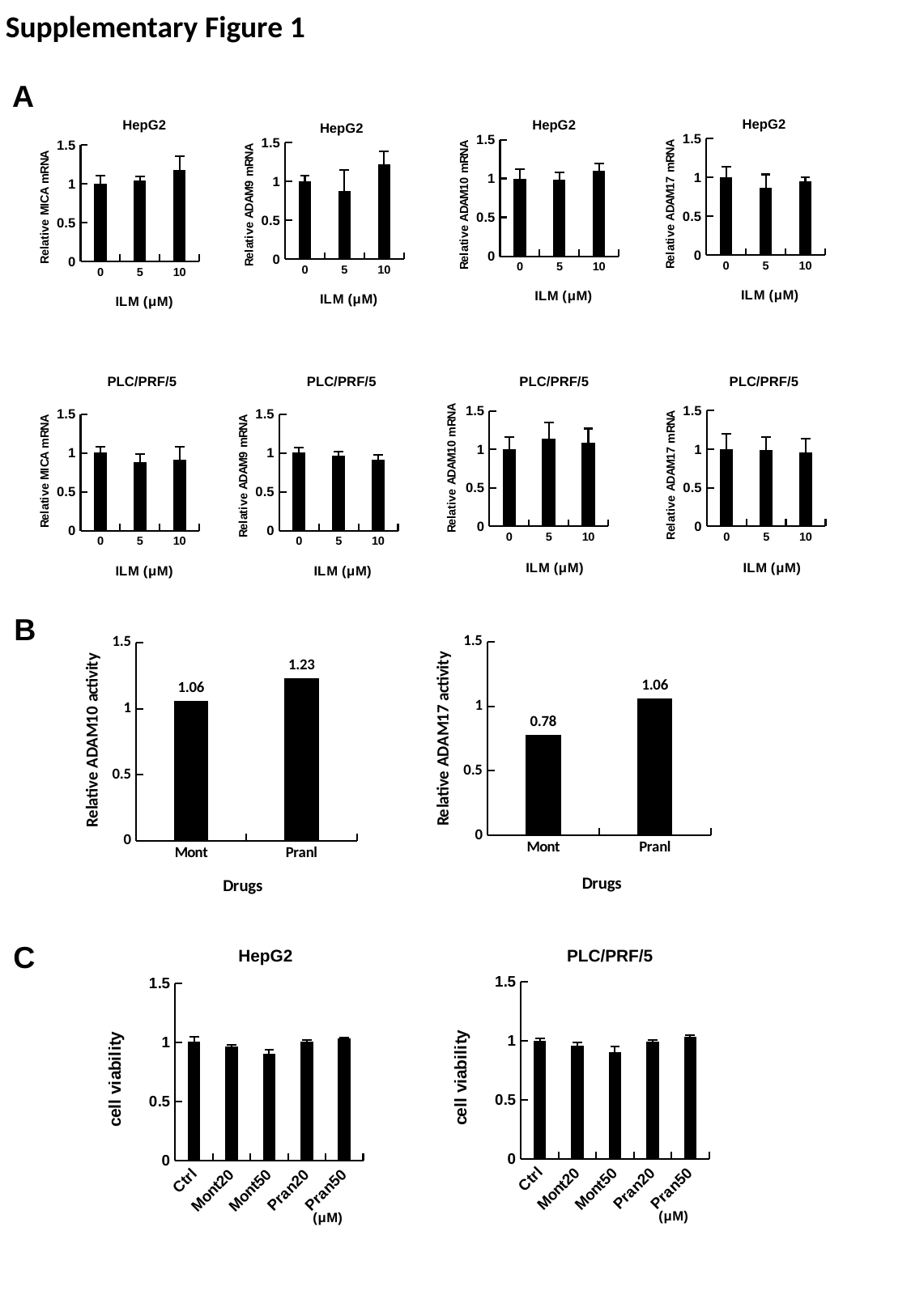

Supplementary Figure 1
A
HepG2
HepG2
HepG2
HepG2
### Chart
| Category | |
|---|---|
| 0 | 1.0 |
| 5 | 0.87 |
| 10 | 0.95 |
### Chart
| Category | |
|---|---|
| 0 | 1.0 |
| 5 | 0.98 |
| 10 | 1.1 |
### Chart
| Category | |
|---|---|
| 0 | 1.0 |
| 5 | 0.88 |
| 10 | 1.22 |
### Chart
| Category | |
|---|---|
| 0 | 1.0 |
| 5 | 1.04 |
| 10 | 1.18 |PLC/PRF/5
PLC/PRF/5
PLC/PRF/5
PLC/PRF/5
### Chart
| Category | |
|---|---|
| 0 | 1.0 |
| 5 | 0.99 |
| 10 | 0.96 |
### Chart
| Category | |
|---|---|
| 0 | 1.0 |
| 5 | 1.13 |
| 10 | 1.08 |
### Chart
| Category | |
|---|---|
| 0 | 1.0 |
| 5 | 0.88 |
| 10 | 0.91 |
### Chart
| Category | |
|---|---|
| 0 | 1.0 |
| 5 | 0.96 |
| 10 | 0.91 |B
### Chart
| Category | |
|---|---|
| Mont | 0.78 |
| Pranl | 1.06 |
### Chart
| Category | |
|---|---|
| Mont | 1.06 |
| Pranl | 1.23 |C
HepG2
PLC/PRF/5
### Chart
| Category | Ctrl |
|---|---|
| Ctrl | 1.0 |
| Mont20 | 0.96 |
| Mont50 | 0.9 |
| Pran20 | 0.99 |
| Pran50 | 1.03 |
### Chart
| Category | Ctrl |
|---|---|
| Ctrl | 1.0 |
| Mont20 | 0.96 |
| Mont50 | 0.9 |
| Pran20 | 1.0 |
| Pran50 | 1.03 |

## Slide 2
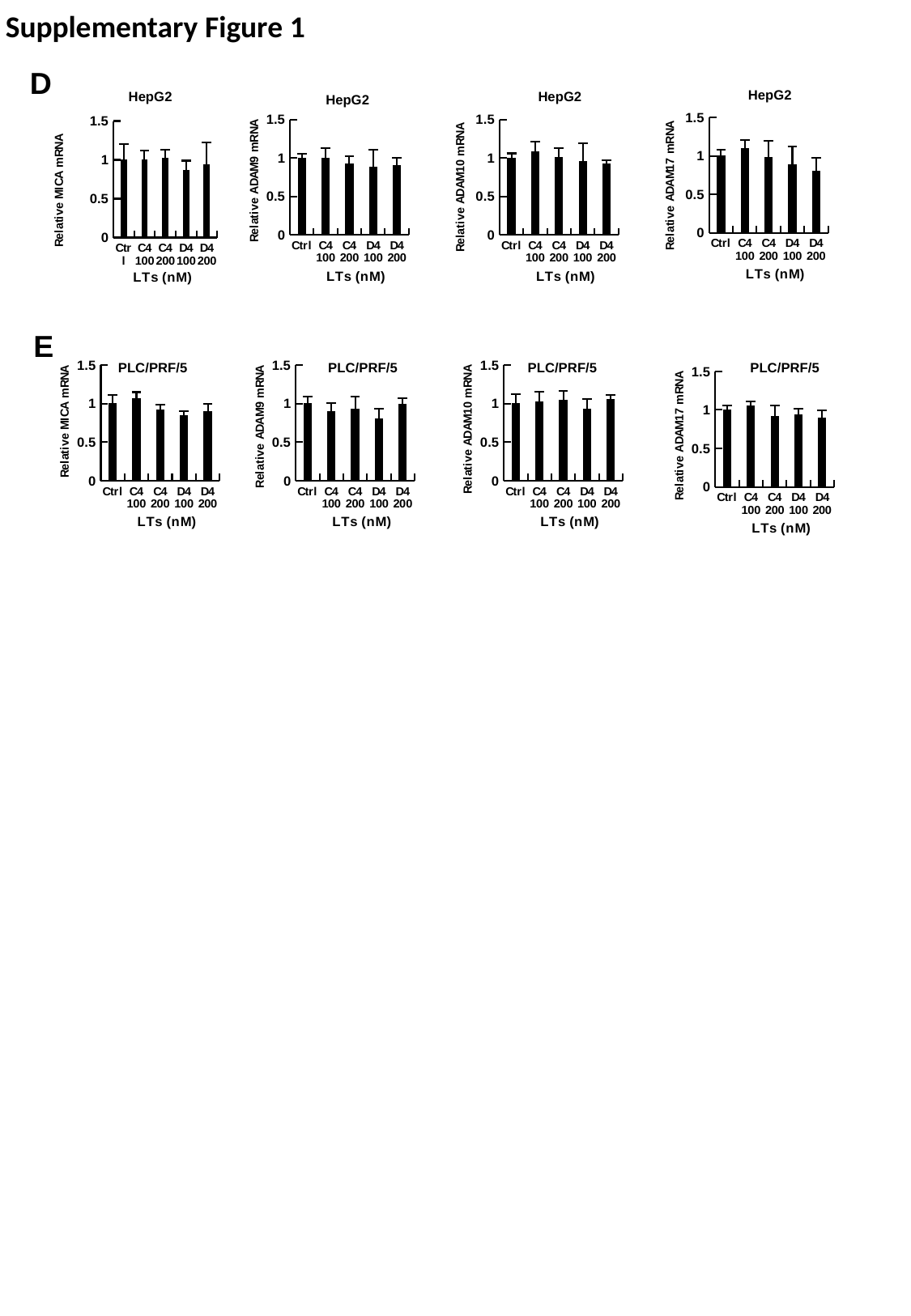

Supplementary Figure 1
D
HepG2
HepG2
HepG2
HepG2
### Chart
| Category | |
|---|---|
| Ctrl | 1.0 |
| C4 100 | 1.1 |
| C4 200 | 0.98 |
| D4 100 | 0.88 |
| D4 200 | 0.8 |
### Chart
| Category | |
|---|---|
| Ctrl | 1.0 |
| C4 100 | 1.0 |
| C4 200 | 0.92 |
| D4 100 | 0.88 |
| D4 200 | 0.9 |
### Chart
| Category | |
|---|---|
| Ctrl | 1.0 |
| C4 100 | 1.08 |
| C4 200 | 1.01 |
| D4 100 | 0.95 |
| D4 200 | 0.92 |
### Chart
| Category | |
|---|---|
| Ctrl | 1.0 |
| C4 100 | 1.0 |
| C4 200 | 1.03 |
| D4 100 | 0.87 |
| D4 200 | 0.94 |E
### Chart
| Category | |
|---|---|
| Ctrl | 1.0 |
| C4 100 | 1.07 |
| C4 200 | 0.92 |
| D4 100 | 0.84 |
| D4 200 | 0.9 |
### Chart
| Category | |
|---|---|
| Ctrl | 1.0 |
| C4 100 | 0.9 |
| C4 200 | 0.93 |
| D4 100 | 0.8 |
| D4 200 | 0.99 |
### Chart
| Category | |
|---|---|
| Ctrl | 1.0 |
| C4 100 | 1.02 |
| C4 200 | 1.04 |
| D4 100 | 0.93 |
| D4 200 | 1.05 |PLC/PRF/5
PLC/PRF/5
PLC/PRF/5
### Chart
| Category | |
|---|---|
| Ctrl | 1.0 |
| C4 100 | 1.05 |
| C4 200 | 0.91 |
| D4 100 | 0.94 |
| D4 200 | 0.89 |PLC/PRF/5
