# Supplementary material for: Leukotriene receptor antagonists enhance HCC treatment efficacy by inhibiting ADAMs and suppressing MICA shedding
Source: Cancer Immunol Immunother. 2020 Jul 18;70(1):203–13. doi: 10.1007/s00262-020-02660-2 (PMC7838147; doi:10.1007/s00262-020-02660-2)
Supplement: Supplementary file 2 — Supplementary file2 (PDF 432 kb) [file 262_2020_2660_MOESM2_ESM.pdf]

January 30, 2018

BEX CO., LTD.
2-61-14 Itabashi Itabashi-ku, Tokyo, Japan
TEL : +81-3-5375-1071
FAX : +81-3-5375-5636

Cell Line Authentication

Client : Jun Arai
Department of Gastroenterology and Hepatology, Showa University

Analysis conducted by Katsunori Imai

Sample：

1. PLC/PRF/5
2. HepG2

Reagent:
GenePrint® 10 System (Promega)

Result:

1. PLC/PRF/5

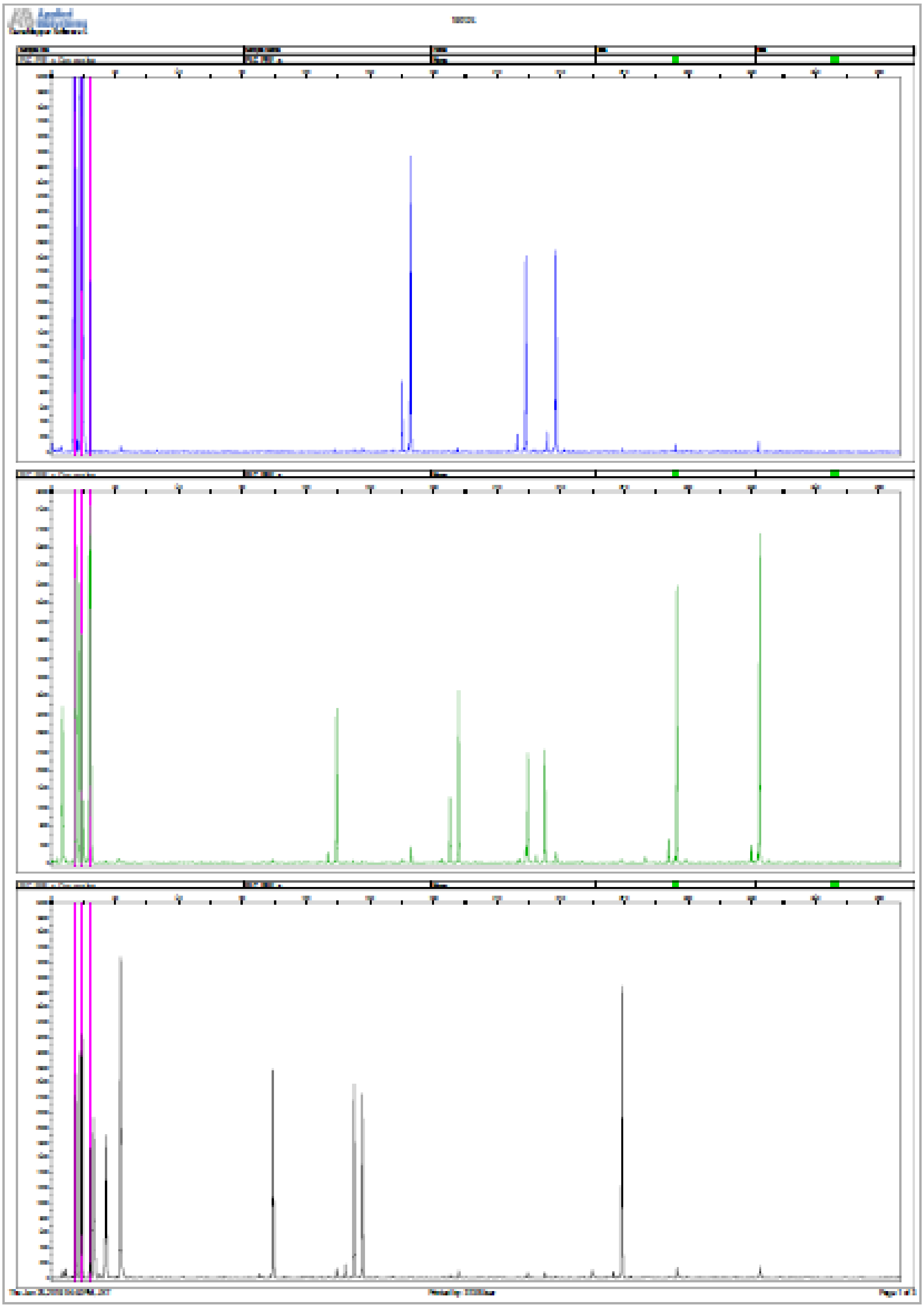

STR Profile

Locus	PLC/PRF/5	
TH01	7	8
D21S11	30	33.2
D5S818	12	
D13S317	11	12
D7S820	9	11
D16S539	13	
CSF1PO	10	
AMEL	X	
vWA	15	16
TPOX	8	

PLC/PRF/5 (ATCC® CRL-8024 ™)	
8	
－	－
12	
11	12
9	11
13	
10	
X	
15	16
8	

STR profiles of PLC/PRF/5 were not completely matched with PLC/PRF/5 (ATCC® CRL-8024™). But Evaluation value (EV) between PLC/PRF/5 and PLC/PRF/5 (ATCC® CRL-8024™) was 0.96, which was high enough that STR profiles of PLC/PRF/5 are the same as those of PLC/PRF/5 (ATCC® CRL-8024™).
Therefore two cell lines were considered to be the identical cell strain.

Reference:
Cell line individualization by STR multiplex system in the cell bank found cross-contamination between ECV304, and EJ-1/T24. Tissue Culture Research Communications,18:329-338(1999)
Tanabe,H., Takada,Y., Minegishi,D., Kurematsu,M. Masui,T., and Mizusawa,H.

2. HepG2

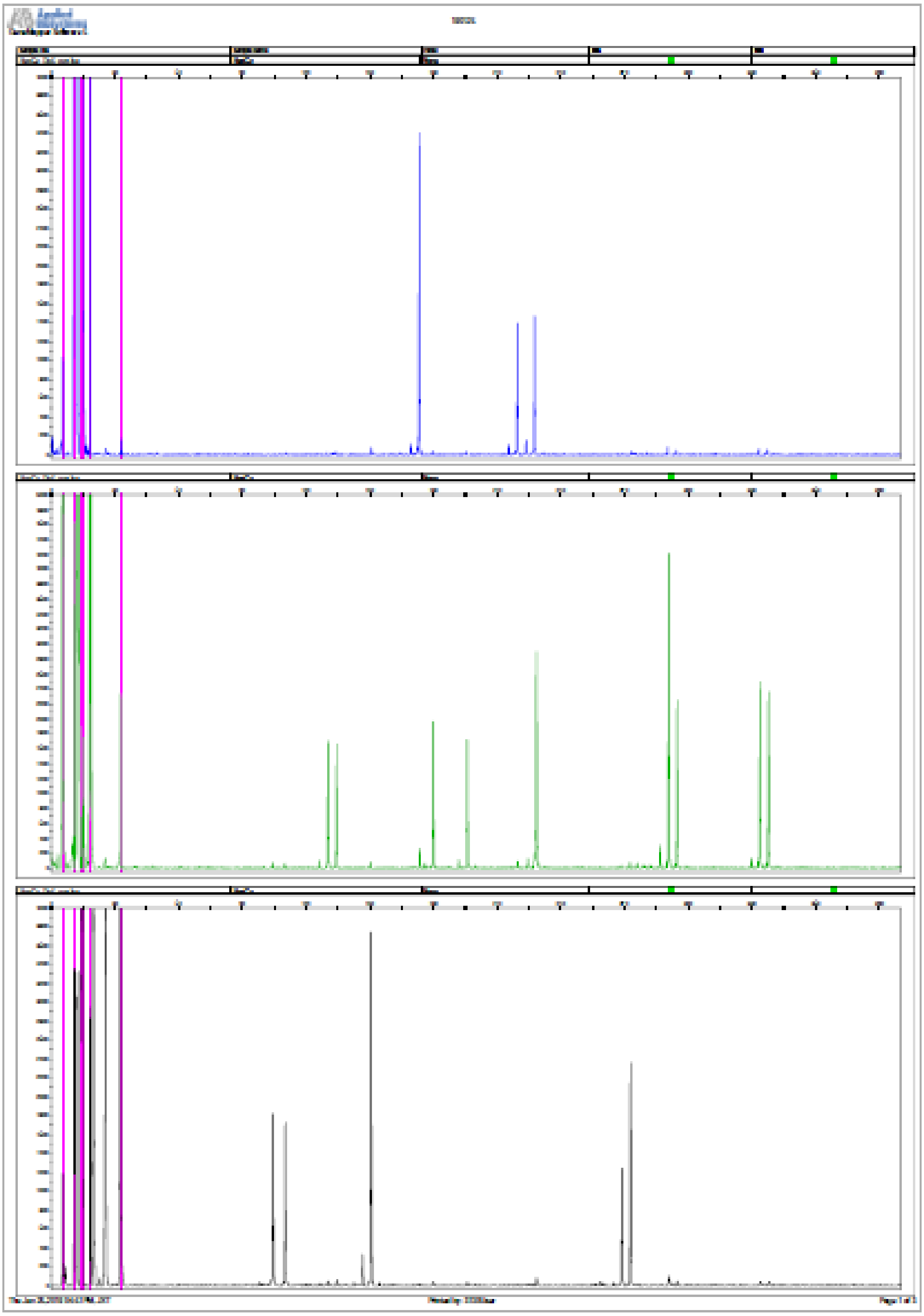

STR Profile

Locus	HepG2		Hep G2 [HEPG2] (ATCC® HB-8065™)	
TH01	9		9	
D21S11	29	31	−	−
D5S818	11	12	11	12
D13S317	9	13	9	13
D7S820	10		10	
D16S539	12	13	12	13
CSF1PO	10	11	10	11
AMEL	X	Y	X	Y
vWA	17		17	
TPOX	8	9	8	9

STR profiles of HepG2 and Hep G2 [HEPG2] (ATCC® HB-8065™) were completely matched. It was verified that the cells analyzed were considered to be the same as the cells registered in ATCC by comparison with the database of ATCC.
