# Supplementary material for: Leukotriene receptor antagonists enhance HCC treatment efficacy by inhibiting ADAMs and suppressing MICA shedding
Source: Cancer Immunol Immunother. 2020 Jul 18;70(1):203–13. doi: 10.1007/s00262-020-02660-2 (PMC7838147; doi:10.1007/s00262-020-02660-2)
Supplement: Supplementary file 3 — Supplementary file3 (PDF 69 kb) [file 262_2020_2660_MOESM3_ESM.pdf]

Supplementary Table 1: Statistical significance in flow cytometry assays

Sample	MFI*	*P*#
HepG2		
NT, IgG	4.39 ± 0.21	0.0022
NT, Anti-MICA	5.86 ± 0.29	-
Montelukast, Anti-MICA	7.06 ± 0.43	0.0070
Pranlukast, Anti-MICA	8.48 ± 0.40	<0.0001
PLC/PRF/5		
NT, IgG	4.33 ± 0.36	0.0034
NT, Anti-MICA	7.40 ± 0.56	-
Montelukast, Anti-MICA	7.51 ± 1.17	0.7871
Pranlukast, Anti-MICA†	9.05 ± 1.02	0.7105

*The average MFIs of three independent flow cytometric assays.
#Comparisons with NT/αMICA as the control using Dunnett’s method.
†Two independent assays were conducted.
