# Supplementary material for: Leukotriene receptor antagonists enhance HCC treatment efficacy by inhibiting ADAMs and suppressing MICA shedding
Source: Cancer Immunol Immunother. 2020 Jul 18;70(1):203–13. doi: 10.1007/s00262-020-02660-2 (PMC7838147; doi:10.1007/s00262-020-02660-2)
Supplement: Supplementary file 4 — Supplementary file4 (PDF 25 kb) [file 262_2020_2660_MOESM4_ESM.pdf]

Supplementary Table 2: Statistical information of flow cytometry

Sample	Mean	Median	CV (%)
Figure 1C			
siCtrl/IgG	7.34	4.87	157.98
siCtrl/αMICA	10.75	7.30	107.36
siADAM9/αMICA	16.36	9.91	109.61
Figure 2D, HepG2			
NT/IgG	4.11	3.31	76.71
NT/αMICA	4.86	3.62	103.52
**Montelukast**_50/αMICA	5.67	4.18	109.70
**Pranlukast** _50/αMICA	5.98	4.26	121.66
Figure 2D, PLC/PRF/5			
NT/IgG	4.33	3.55	71.94
NT/αMICA	7.40	4.61	113.82
**Montelukast** _50/αMICA	7.51	4.96	106.24
**Pranlukast** _50/αMICA	9.05	5.09	111.87
Figure 3F			
NT/IgG	4.74	3.55	116.23
NT/αMICA	8.67	5.52	119.40
siCtrl/ **leukotriene** D4_100 /αMICA	8.22	4.70	167.20
siADAM9/ **leukotriene** D4_100 /αMICA	8.89	5.52	126.67
Figure 4A, SOR/ **leukotriene receptor antagonists**			
NT/IgG	4.34	3.55	81.08
NT/αMICA	7.03	5.19	101.20
SOR/αMICA	7.01	5.42	624.65
SOR/ **montelukast** /αMICA	10.08	6.85	444.48
SOR/ **pranlukast** /αMICA	11.47	7.37	104.72
Figure 4A, REG/ **leukotriene receptor antagonists**			
NT/IgG	4.42	3.55	116.23
NT/αMICA	7.07	5.23	99.84
REG/αMICA	7.79	5.94	556.54
REG/ **montelukast** /αMICA	8.70	6.04	97.24
REG/ **pranlukast** /αMICA	11.78	7.84	100.30
