# Supplementary material for: Leukotriene receptor antagonists enhance HCC treatment efficacy by inhibiting ADAMs and suppressing MICA shedding
Source: Cancer Immunol Immunother. 2020 Jul 18;70(1):203–13. doi: 10.1007/s00262-020-02660-2 (PMC7838147; doi:10.1007/s00262-020-02660-2)
Supplement: Supplementary file 6 — Supplementary file6 (DOCX 29 kb) [file 262_2020_2660_MOESM6_ESM.docx]

**Supporting Information of Supplementary Table Legends**

**Supplementary Table 1.**

Statistical significance in flow cytometry assays.

**Supplementary Table 2.**

Changes in transcriptional levels and enzymatic activity of MICA and ADAM after treatment with montelukast/pranlukast. The arrows ↑, ↓ indicate a significant increase or decrease compared to the untreated cells, respectively. *P*-values less than 0.05 were considered statistically significant. → indicates no significant difference.

**Supplementary Table 3.**

Statistical information of flow cytometry is shown. SOR: sorafenib, REG: Regorafenib

**Supplementary Figure 1*.***

1. Relative mRNA levels of MICA and ADAMs were analyzed by qRT-PCR after ilomastat treatment.
2. Enzymatic inhibition of ADAM10 and ADAM17 by pranlukast or montelukast *in vitro*.
3. Cell viabilities were determined using CCK8 assay after treatment with pranlukast and montelukast in HepG2 and PLC/PRF/5 cells.

Relative mRNA levels of MICA and ADAMs were analyzed using qRT-PCR after treating the HepG2 (D) and PLC/PRF/5 cells (E) with leukotriene C4/D4. ILM: ilomastat
